# Negative pressure pulmonary edema postextubation following medial nerve repair with sural graft surgery in a young patient: A case report

**DOI:** 10.1097/MD.0000000000013743

**Published:** 2018-12-28

**Authors:** Byron Rosero-Britton, Alberto Uribe, Nicoleta Stoicea, Luis Periel, Sergio D. Bergese

**Affiliations:** a Grupo de Investigaciones Básicas y Clínicas, Gibacus, Universidad del Sinú, Cartagena; b Department of Anesthesiology, Hospital Universitario de San José, Bogota, Colombia; c The Ohio State University Wexner Medical Center, Department of Anesthesiology; d The Ohio State University Wexner Medical Center, Department of Neurological Surgery, Columbus, OH.

**Keywords:** anesthesia, extubation, negative pressure pulmonary edema, postoperative pulmonary complications

## Abstract

**Rationale:**

Negative pressure pulmonary edema (NPPE) is a serious well-described pulmonary complication. It occurs after an intense inspiratory effort against an obstructed or closed upper airway and generates a large negative airway pressure, leading to severe pulmonary edema (transvascular fluid filtration and interstitial/alveolar edema) and hypoxemia. We present a case of NPPE following general anesthesia in a patient who underwent median nerve neurorrhaphy with graft from lower left limb (sural nerve) due to sharp injury.

**Patient concerns:**

A 39-year-old Hispanic male was admitted to the Hospital Universitario de San José and scheduled to undergo a median nerve neurorrhaphy under general anesthesia. Preoperative vital signs, physical examination, and laboratory assessments were unremarkable. At the end of surgery, anesthetic agents were ceased after patient responded to commands and maintained eye contact. However, immediately after extubation, anesthesia care providers observed marked respiratory distress and rapid development of hypoxia.

**Diagnoses:**

After extubation, patient presented multiple episodes of hemoptysis, tachypnea (25 per minute), blood oxygen saturation (SpO_2_) of 82% and abundant bilateral pulmonary rales. A baseline chest x-ray revealed symmetric parenchymal opacities with ground-glass attenuation and bilateral multilobar consolidations patterns. The diagnosis of NPPE was established and supportive treatment was initiated.

**Interventions:**

The patient received noninvasive mechanical ventilation with a PEEP at 10 cmH_2_O, intravenous furosemide (20 mg.) every 12 hours, and fluids restriction. Patient remained in PACU for continuing monitoring and laboratory/imaging follow-up testing until next morning.

**Outcomes:**

On postoperative day 1, patient responded satisfactorily to supportive treatment and transferred to the general care floor; oxygen supplementation was discontinued 12 hours after extubation time. On postoperative day 3, after the evaluation of a chest x-ray, patient was discharged to home in stable conditions

**Lesson:**

The occurrence of NPPE in the perioperative setting could be successfully managed with supportive regimens, effective clinical team coordination, and awareness of the importance of its rapid diagnosis.

## 1 Introduction

The occurrence of postoperative pulmonary complications (PPCs) is a common concern among surgical and critical care wards due to its association with morbidity, mortality, hospital and intensive care unit (ICU) readmission, prolonged hospital stay, and financial burden.^[1–5]^ The incidence of PPCs reported in the literature varies between 9% and 40% due to its association with different types of surgery, quantification of respiratory problems and other perioperative patient-related variables.^[3,4,6]^

Negative pressure pulmonary edema (NPPE) is a serious well-described pulmonary complication.^[7–11]^ The occurrence of NPPE is rare; the incidence ranges from 0.01% to 0.05% among all anesthetic procedures and 4% of all laryngospasm events.^[12,13]^ It occurs after an intense inspiratory effort against an obstructed or closed upper airway and generates a large negative airway pressure, leading to severe pulmonary edema (transvascular fluid filtration and interstitial/alveolar edema) and hypoxemia.^[7–11,14]^ The most common cause (50%) of upper airway obstruction documented in cases of NPPE is the laryngospasm; it usually starts with an isolated respiratory failure event that occurs during intubation or immediately after extubation or laryngeal mask removal.^[12]^ Two types of NPPE have been described in the literature: type I occurs when there is forced inspiration in the presence of acute obstruction of the airway,^[11,15]^ while type II occurs after releasing a chronic airway obstruction (resection of laryngeal tumor and intrathoracic lesions).^[9,10]^ There is not a strong consensus of the predictors for this complication, but the risk factors associated with NPPE include, but are not limited to obesity, short neck, acromegaly, obstructive sleep apnea, and upper airway surgery.^[12,16]^ In addition, NPPE is prevalent in young, healthy and muscular patients with the ability and strength to create higher negative pressures.^[9,17]^ The treatment of NPPE is supportive depending on the severity of the symptoms: it includes oxygen supplement to demand and positive end-pulmonary pressure in critical cases.^[8,12,18,19]^

## 2 Case description

A 39-year-old Hispanic male, 69 kg, 170 cm (body mass index: 23,), American Society of Anesthesiologist physical status (ASA) I, with a history of untreated peptic ulcer disease and appendectomy (10 years ago) was admitted to the hospital and scheduled to undergo median nerve neurorrhaphy with graft from lower left limb (sural nerve) due to sharp injury under general anesthesia. Preoperative vital signs, physical examination and laboratory assessments were unremarkable. Patient's verbal consent was obtained in order to publish the case report with de-identified data as per institutional standard procedures.

Conventional intraoperative monitoring (electrocardiogram, noninvasive blood pressure, and peripheral oxygen saturation) and intravenous access for fluid/drug administration were performed per institutional routine. During anesthesia induction, the patient in supine position received intravenous propofol (140 mg), cisatracurium (6 mg), and remifentanil 0.5 mcg/kg/min. After loss of consciousness confirmation, a single endotracheal intubation attempt with an 8.0 tube was performed.

Anesthesia maintenance was achieved with isoflurane 0.6 mean alveolar concentration (MAC) and intravenous remifentanil at 0.3 mcg/kg/min. Ventilator mode consisted on continuous mandatory ventilation (CMV) 500 mL, positive end expiratory pressure (PEEP) at 5 cmH_2_O, respiratory rate (RR) was 10/min, and ratio 1:2. The length of surgery was 150 minutes; the patient remained thermodynamically stable in supine position with 105-minutes tourniquet application on left arm. Intravenous dexamethasone (8 mg) and metoclopramide (10 mg) was administered as antiemetic prophylaxis. Analgesia was managed with intravenous morphine (6 mg). A total of 1500 mL of lactate ringer was infused during surgery.

**Figure 1:**
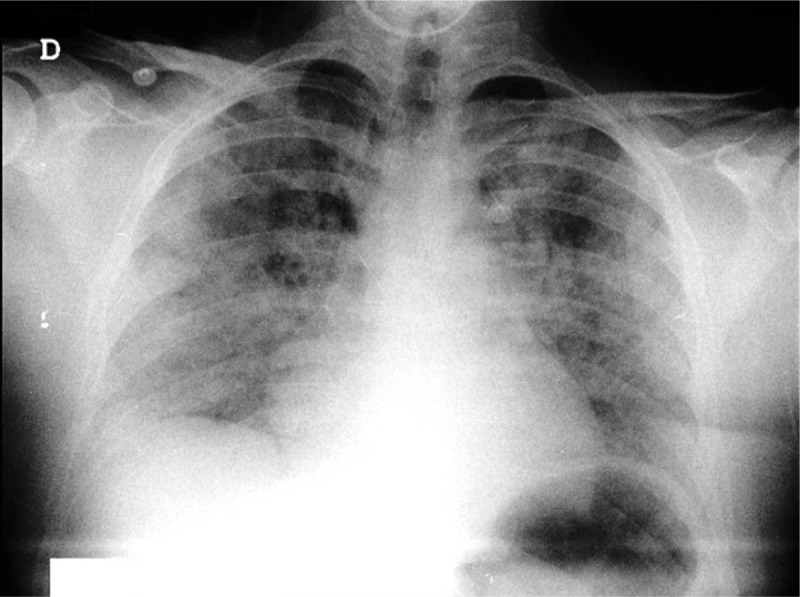
Chest radiograph upon PACU admission. PACU = postanesthesia care unit.

At the end of surgery, anesthetic agents were ceased and after patient responded to commands, maintained eye contact, and a TOF (Train-Of-Four) of 94% was documented, the endotracheal extubation was performed. However, immediately after extubation, anesthesia care providers observed marked respiratory distress, inspiratory effort, wheezing, cyanosis and rapid development of hypoxia with oxygen saturation level (SpO_2_) below 90% for approximately 30 seconds. Therefore, immediate positive pressure mask ventilation with 100% fraction of inspired oxygen (FiO_2_) was started and improved patient clinical status one minute after. Consequently, he was transferred to the postanesthesia care unit (PACU) with normal vital signs and no signs of respiratory distress. Nevertheless, 15 minutes after PACU admission, patient presented multiple episodes of hemoptysis, tachypnea (25 per minute), SpO_2_ of 82% and abundant bilateral pulmonary rales. Arterial blood gas revealed acute metabolic acidosis and included pH 7.33, partial pressure of oxygen (PaO_2_) 44.4 mm Hg, partial pressure of carbon dioxide (PaCO_2_) 40.0 mm Hg, bicarbonate (HCO3) 20.5 mmol/L, base excess −5.5 mmoL and oxygen saturation of 79.3%, lactate 2.4 mmol/L. A baseline chest x-ray revealed symmetric parenchymal opacities with ground-glass attenuation and bilateral multilobar consolidations patterns (Fig. 1).

Based on the aforementioned information, a diagnosis of NPPE was established and supportive treatment was immediately initiated with noninvasive mechanical ventilation with a PEEP at 10cmH2O, intravenous furosemide (20 mg) every 12 hours, and fluids restriction. Patient remained in PACU for continuing monitoring and laboratory/imaging follow-up testing until next morning.

On postoperative day 1, patient responded satisfactory to supportive treatment; his arterial blood gas revealed a pH 7.43, PaO_2_ 58.6 mm Hg, PaCO_2_ 39.0 mm Hg, HCO_3_ 25.3 mmol/L, base excess 1.0 mmoL and oxygen saturation of 92.8%; therefore, patient was transferred to the general care floor requiring no oxygen supplementation 12 hours after extubation time.

**Figure 2:**
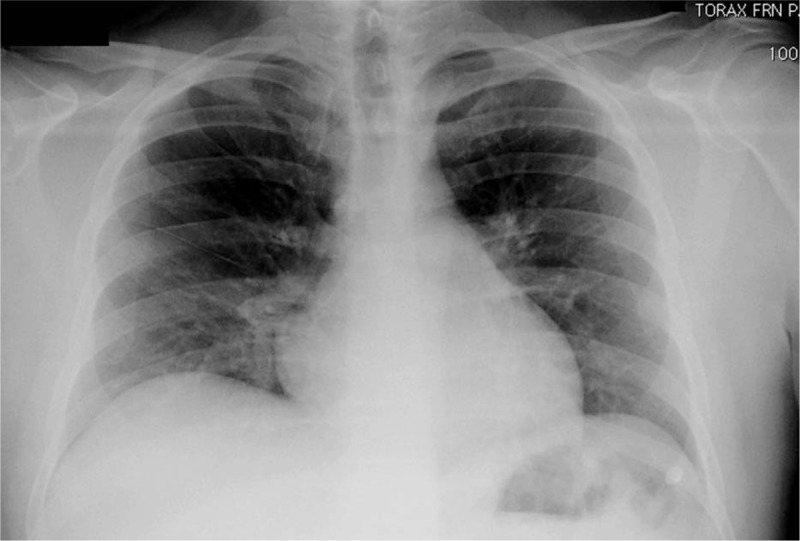
Chest radiograph on postoperative day 3.

Subsequently, on postoperative day 3, patient was discharged to home in stable conditions after the evaluation of a chest x-ray that revealed right basal parenchymal opacity related to atelectasis with non-clinical significance (Fig. 2).

## 3 Discussion

We described a case of pulmonary edema due to negative pressure after endotracheal extubation. The classic clinical evolution of NPPE begins immediately after the emergence phase of general anesthesia; however a late onset (up to 24 hours) has been reported in the literature, so patients presenting with laryngospasm should be monitored for up to 24 hours after the emergence from general anesthesia.^[20]^ A rapid diagnosis and intervention is required for NPPE as it could eventually lead to acute severe hypoxemia.^[21,22]^ For this particular patient, Type 1 NPEE was promptly determined which is usually the result of forced inspiration against a partially obstructed or closed glottis, creating an excessive decrease in intrathoracic pressure of approximately −100 to −140 cmH_2_O (normal value: −4 cmH_2_O).^[23,24]^ This causes an increase in venous return to the right side of the heart (preload), producing an increase in the hydrostatic pressure of the pulmonary venous circulation, which generates a trans-pulmonary hydrostatic gradient with movement from a space with high pressure (pulmonary capillaries) to one with low pressure (pulmonary and alveolar interstitium) and disruption of alveolar capillary membrane.^[25]^ Consequently, gas exchange decreases producing hypoxemia, catecholamine release, and systemic and pulmonary hypertension.^[26]^ The degree of “alveolar flooding” depends on the extent of interstitial edema, the presence or absence of alveolar epithelial lesion or the ability of the alveolar epithelium to actively remove the fluid.^[27]^ Identification of abundant rales in pulmonary fields, tachypnea, and gradual oxygen desaturation were all symptoms and signs that are consistent with NPPE diagnosis.^[21]^ Patient's risk factors are his relatively young age and his muscular appearance.^[23]^ Noninvasive respiratory support performed in this case represents the common treatment for NPPE patients and it is used as a valid approach by other recent studies.^[28–30]^ Focusing on the prevention and early identification of postoperative laryngospasm could further lower the incidence of NPPE. Prevention usually involves lidocaine topical administration or laryngotracheal anesthesia, use of throat packs and cautious oropharyngeal suction in order to reduce stimulation/manipulation and irritation of the larynx.^[16]^

## 4 Conclusion

In conclusion, despite NPPE being reported in the literature as a self-limited event with favorable prognosis and simple management, its prevention and/or early identification diminishes the financial burden and the severity of clinical outcomes with the avoidance of prolonged hospitalizations or intensive care unit admissions. The occurrence of NPPE in the operating room could be managed successfully with supportive regimens, effective clinical team coordination, and awareness of the importance of its rapid diagnosis. Potential risk factors and accessible preventive methods for patients undergoing surgery should be considered, in order to maximize safety in surgical and intensive care settings.

## Author contributions

**Conceptualization:** Alberto Uribe, Byron Rosero-Britton.

**Data curation:** Alberto Uribe, Byron Rosero-Britton.

**Formal analysis:** Alberto Uribe, Byron Rosero-Britton.

**Investigation:** Alberto Uribe, Byron Rosero-Britton.

**Methodology:** Alberto Uribe, Byron Rosero-Britton, Nicoleta Stoicea.

**Project administration:** Alberto Uribe, Byron Rosero-Britton, Nicoleta Stoicea.

**Resources:** Byron Rosero-Britton.

**Supervision:** Alberto Uribe, Byron Rosero-Britton, Nicoleta Stoicea, Sergio D. Bergese.

**Validation:** Alberto Uribe, Byron Rosero-Britton, Nicoleta Stoicea, Sergio D. Bergese.

**Visualization:** Alberto Uribe, Byron Rosero-Britton.

**Writing – original draft:** Alberto Uribe, Byron Rosero-Britton, Nicoleta Stoicea, Luis Periel, Sergio D. Bergese.

**Writing – review & editing:** Alberto Uribe, Byron Rosero-Britton, Nicoleta Stoicea, Luis Periel, Sergio D. Bergese.

Alberto Uribe orcid: 0000-0001-7897-8322.
